# Natural Orifice Translumenal Endoscopic Surgery: Review of Its Applications in Bariatric Procedures

**DOI:** 10.1007/s11695-015-1978-y

**Published:** 2015-11-30

**Authors:** Simon Erridge, Mikael H. Sodergren, Ara Darzi, Sanjay Purkayastha

**Affiliations:** Department of Surgery and Cancer, Imperial College London, London, UK

**Keywords:** NOTES, Natural orifice, Gastrectomy, Sleeve, Endolumenal

## Abstract

This review aims to summarise clinical applications of natural orifice translumenal endoscopic surgery (NOTES) in bariatric surgery. A review of data, until December 2014, was carried out regarding techniques and outcomes of bariatric NOTES procedures. Nine publications were included in the final analysis, with another six papers describing endolumenal procedures included for comparison. All NOTES studies adopted a hybrid procedure. Hybrid NOTES sleeve gastrectomy (hNSG) was described in four humans and two porcine studies. In humans, six subjects (23.1 %) were converted to conventional laparoscopic methods, and one postoperative complication (3.8 %) was reported. Mean excess weight loss was 46.6 % (range 35.2–58.9). Transvaginal-assisted sleeve gastrectomy appears feasible and safe when performed by appropriately trained professionals. However, improvements must be made to overcome current technical limitations.

## Introduction

Bariatric surgery aims to reduce comorbidities and limit long-term health implications in obese patients including diabetes mellitus, hypertension, hyperlipidaemia and obstructive sleep apnoea. There is now a significant body of evidence supporting the benefits of bariatric surgery in regard to both weight loss and reduction of comorbidities [1–3].

Similarly, there is evidence from other interventions to support further minimisation beyond conventional laparoscopic techniques using single-incision laparoscopic surgery (SILS). Evidence regarding SILS cholecystectomy morbidity is favourable [4], and there is some indication the SILS approach provides improved cosmesis compared to current laparoscopic practice [5]. Data regarding SILS bariatric procedures is scarce. Despite this, there is data to support the use of SILS over multiple ports in a select group of patients [6].

Natural orifice translumenal endoscopic surgery (NOTES) aims to further reduce the invasiveness of surgery through minimisation of abdominal trauma. A ‘pure NOTES’ procedure prevents incision of the abdominal wall, and hybrid NOTES procedures minimise the number of port sites in the abdomen. This is especially pertinent in a sleeve gastrectomy (SG), where one of the port sites has to be enlarged for removal of the gastric remnant.

There is some evidence to suggest that NOTES has clinical advantages in other applications. A recent meta-analysis by Sodergren et al. shows that transvaginal hybrid NOTES cholecystectomies lead to a reduction in postoperative pain and a faster return to normal activities [7].

There is a paucity of data on NOTES’ application in bariatric procedures. This study aims to review currently published literature in relation to bariatric NOTES applications (including endolumenal procedures) in terms of clinical outcomes, as well as variations in surgical techniques and to provide guidance on current practices as well as future areas for research.

## Methods

A narrative review of the available literature was undertaken. For this, a broad search of the literature was conducted using PubMed and Google Scholar. The keywords for the search included the following: ‘transvaginal’, ‘NOTES’, ‘sleeve’, ‘bariatric’, ‘obesity’, ‘natural orifice’, ‘bypass’, ‘rny’, ‘lrygb’, ‘mgb’ and ‘bpd’. To widen the search, the bibliographies of all publications were searched for relevant references. Studies were included up to and including December 2014. The inclusion criteria specified all articles related to a natural orifice translumenal endoscopic approach to bariatric procedures in human, cadaveric or animal subjects.

Data extracted took the form of both clinical outcomes and operative technique. Outcomes that were observed included mean operative time, mean hospital stay, mean excess weight loss and mean operative time. As much relevant data as possible was extracted in regard to operative technique.

## Results

A total of nine studies [8–16], describing bariatric NOTES procedures in 29 humans, 17 animals and 8 cadavers, were included in this review. Table 1 displays the three operations described, the number of endoscopes used and the translumenal route. All studies favoured the use of a hybrid approach over a pure NOTES procedure.

**Table 1:** Studies included in review

Author	Year of publication	Subjects	Number of subjects	Operation	Number of endoscopes used	Endoscopic route
Chouillard et al. [8]	2011	Human	20	hNSG	1	TV
Fischer et al. [9]	2009	Human	1	hNSG	1	TV
Lacy et al. [11]	2009	Human	1	hNSG	1	TV
Ramos et al. [16]	2008	Human	4	hNSG	1	TV
Marchesini et al. [13]	2008	Porcine	12	hNSG	1	TV
Mintz et al. [15]	2008	Porcine	5	hNSG	2	TR + TG
Michalik et al. [14]	2011	Human	3	hNAGB	1	TV
Hagen et al. [10]	2008	Cadaveric	7	hNRYGB	2	TV + TG
Madan et al. [12]	2008	Cadaveric	1	hNRYGB	2	TV + TG

*hNSG* hybrid NOTES sleeve gastrectomy, *hNAGB* hybrid NOTES adjustable gastric band, *hNRYGB* hybrid NOTES Roux-en-Y gastric bypass, *TV* transvaginal, *TR* transrectal, *TG* transgastric

### Hybrid NOTES Sleeve Gastrectomy

Four humans and two porcine studies described hybrid NOTES sleeve gastrectomy (hNSG) procedures in 26 patients and 17 animals. Only Mintz et al. [15] described an approach other than transvaginal, using one endoscope transrectally and another transgastrically. Table 2 outlines the hNSG procedures performed, providing technical data.

**Table 2:** Hybrid NOTES sleeve gastrectomies performed in humans and animals

Author	Year of publication	Subjects	Number of subjects	Endoscopic route	Number of additional port sites	Mean operative time: min (range)	Postoperative complication rate
Chouillard et al. [8]	2011	Human	14/20 attempted	TV	2	116 (54–231)	1 (5 %)
Fischer et al. [9]	2009	Human	1	TV	1	171	Nil
Lacy et al. [11]	2009	Human	1	TV	4	150	Nil
Ramos et al. [16]	2008	Human	4	TV	3	95 (90–100)	Nil
Marchesini et al. [13]	2008	Porcine	12	TV	3	84	Nil
Mintz et al. [15]	2008	Porcine	5	TR + TG	1	N/A (150–300)	Nil

### Clinical Outcomes

#### Human Studies

Despite all operations being successful, six human subjects (23.1 %) had to be converted to a conventional laparoscopic technique. Of these, four were due to poor visualisation and two were due to bleeding [8]. Mean operative time in humans was 113 min (range 54–231). It is unclear as to whether this adjusted to include the cases in which conversion was necessary.

Mean length of hospital stay was 67 h ranging from 24–144 h.

Mean excess weight loss was described in two studies [8, 9] with 21 human subjects. At 6 months, there was a mean excess weight loss in these patients of 46.6 % (range 35.2–58.9).

In comparison, the laparoscopic procedure has a reported mean operative time of 86.8 min (range 29–207) with a mean excess weight loss of 45.2 % (range 31.1–71.6) [17].

#### Animal Studies

In regard to animal studies, mean operative time described by Marchesini et al. was 84 min [13], whilst Mintz et al. obtained an operative time range of 150–300 min [15]. One postoperative complication (pneumonia) was reported—a rate of 3.8 %. This was treated effectively by physiotherapy and antibiotics [8]. There were no long-term complications reported at follow-up.

### Variations in Surgical Technique

#### Human Studies

Common to all studies was the positioning of patients [8, 9, 11, 16]. Patients were initially placed in the lithotomy position to make an incision in the vaginal wall before moving to the Trendelenburg position to draw the bowel out of the pelvis for trocar insertion. The patients were placed in the Trendelenburg position for the rest of the operation.

Additionally, each vaginal trocar was inserted under laparoscopic guidance provided through the initial port site, which was also used to maintain pneumoperitoneum [8, 9, 11, 16].

Division of the short gastric vessels was attempted endoscopically in two cases [8, 9]. However, in both studies, the initial port site was also used in freeing the greater curvature. In the case of Chouillard et al., the greater curvature was freed whilst using a gastric retractor laparoscopically [8]. Fischer et al. relied on using a harmonic scalpel through vaginal and abdominal ports to reach the oesophageal hiatus [9]. In the other two human studies, division was only attempted via abdominal ports with the endoscope providing visualisation [11, 16].

Chouillard, Lacy and Ramos utilised the umbilical port only for transection using a linear stapler [8, 11, 16] (Table 3). Fisher et al. used a stapler through the LUQ port to transect the stomach; however, the endoscope was utilised to grasp and manipulate the stomach as well as suturing the gastric remnant [9].

**Table 3:** Difference in surgical technique in human studies

Author	Site of initial port (size)	Additional ports (size)	Endoscopic port (size)	Pneumoperitoneum	Division of short gastric vessels	Transection of stomach	Closure
Chouillard et al. [8]	Umbilical (12 mm)	Left flank (5 mm)	TV (15 mm)	Umbilical	Umbilical, TV	Umbilical	Absorbable suture
Fischer et al. [9]	LUQ (12 mm)	Nil	TV (15 mm)	LUQ	LUQ, TV	LUQ	Absorbable suture
Lacy et al. [11]	Umbilical (12 mm)	LUQ (2 mm), RUQ (2 mm), xiphoid (2 mm)	TV (12 mm)	Umbilical	Umbilical	Umbilical	N/A
Ramos et al. [16]	Umbilical (10–12 mm)	RUQ (5 mm), LUQ (2 mm)	TV (12 mm)	Umbilical	Umbilical	Umbilical	Vicryl

There was no uniformity in the size of bougie used with 33, 34, 60 and 36 F being used by Chouillard, Fischer, Lacy and Ramos, respectively [8, 9, 11, 16].

#### Animal Studies

Marchesini et al. [13] used an umbilical port (12 mm) to maintain pneumoperitoneum, guidance of colpotomy, release of greater curvature using ultrasonic scalpel and gastric transection with an endolumenal stapler. Two portals (2 mm) were placed laterally for triangulation. The endoscope, inserted directly through the colpotomy, was used for traction of the greater curvature with endoscopic traction forceps. A 32 F bougie was used in these procedures.

Mintz et al. [15] used an endoscope rectally and a gastroscope, which illuminated the gastro-oesophageal junction. The rectal trocar (12–15 mm) was used for gastric transection with an Endo-GIA stapler and division of short gastric vessels using a harmonic scalpel. An extra-long Endo-GIA together with manipulation of stomach with a gastroscope was necessary to transect up to the gastro-oesophageal junction (GOJ), whilst stapler placement was troubling throughout. An abdominal port (5 mm) was used for retraction and exposure of the stomach using stay sutures.

Common to both human and animal studies was that the gastric remnant was removed through the endoscope insertion site [8, 9, 11, 13, 15, 16].

### Hybrid NOTES Adjustable Gastric Band

Michalik et al. [14] performed a hNAGB procedure on three females. Table 4 gives an outline of the procedure, alongside clinical data.

**Table 4:** Hybrid NOTES adjustable gastric bands inserted in patients

Author	Year of publication	Subjects	Number of subjects	Endoscopic route	Number of additional port sites	Mean operative time: min (range)	Postoperative complication rate
Michalik et al. [14]	2011	Human	2/3 attempted	TV	2	110 (80–145)	1

### Clinical Outcomes

One procedure (33.3 %) was converted to conventional laparoscopic methods due to difficulties positioning and closing the band.

The postoperative complication rate was 33.3 % as one patient suffered right ureteric damage with formation of an uretero-vaginal fistula. This was corrected by a right nephrostomy and subsequent re-implantation of the right ureter into the bladder.

Mean weight loss at 2 months in these patients was 15 kg (range 14–16). This was with an average of 3 ml of fluid injected into the port site. There were no signs of dysphagia.

These data cannot be compared accurately to that relating to laparoscopic procedures as weight loss is normally reported in terms of percentage excess weight loss, as opposed to absolute weight loss. For some contrast, the mean excess weight loss in open and laparoscopic gastric banding at 2 years is 49.4 % reported in a systematic review of the long-term follow-up of bariatric surgery [18].

### Surgical Technique

Michalik et al. [14] used a 5 mm umbilical port for control of the colpotomy in the lithotomy position. A Veress needle in the left hypochondrium established pneumoperitoneum; this was later used for a 15-mm port site. The patient was then moved to the reverse Trendelenburg position. A 10 mm laparoscopic liver retractor was used through the left hypochondrium port, allowing visualisation of the pars flaccida. The endoscope, inserted directly through the colpotomy, was used to dissect the pars flaccida and position the band. This was achieved by passing the endoscope posterior to the stomach, through the pars flaccida to the angle of His to catch the band with endoscopic grasping forceps. The gastric band was inserted through the 15-mm port site. In the two successful cases, the endoscope was able to position the band correctly and close it with assistance from one abdominal port site. The band’s port was embedded through the 15 mm trocar opening.

### Hybrid NOTES Roux-en-Y Gastric Bypass

Two cadaveric studies assessed the feasibility of performing hNRYGB in eight cadavers. For this procedure, it was required that two endoscopes be used, both transvaginally and transgastrically. Table 5 provides a broad outline of the results of these studies.

**Table 5:** Hybrid NOTES Roux-en-Y gastric bypass procedures in cadavers

Author	Year of publication	Subjects	Number of subjects	Endoscopic route	Number of additional port sites	Mean operative time: min (range)
Hagen et al. [10]	2008	Cadaveric	4/7 attempted	TV + TG	2 to 3	N/A (6–9 h)
Madan et al. [12]	2008	Cadaveric	1	TV + TG	2	N/A

### Procedural Outcomes

Unfortunately, in Hagen et al.’s study [10], three procedures could not advance to completion. In one case, this was due to time constraints. In the other two, the cadavers had advanced decay and anatomical difficulties preventing continuation.

Moreover, in forming the anastomosis, there was some difficulty in analysing the length of small bowel [10].

### Surgical Technique

Hagen et al. [10] inserted a 12-mm umbilical port for laparoscopic guidance of vaginal trocar (15 mm) insertion. In forming the gastric pouch, transabdominal stapling was found to be difficult. An intragastric endoscope provided retraction and also measured the length of small bowel. Gastro-entero anastomosis was achieved using a transoesophageal stapler and anvil. Anvil docking was only possible laparoscopically. Enterotomies were more difficult using the transvaginal endoscope. The transvaginal endoscope was used for dissection of the gastric pouch and circular anvil introduction for transoesophageal stapling. Linear laparoscopic staplers were introduced via the vaginal trocar for formation of an entero-entero anastomosis and resection of the immediate part of small bowel.

Madan et al. [12] used a transgastric endoscope via an anterior mid-body gastrotomy to visualise vaginal trocar (12 mm) insertion. Another port was inserted in the right vaginal wall. A trocar was placed in the abdomen for laparoscopic visualisation only. The bowel and mesentery were separated using a linear stapler through the right vaginal port. An enterotomy was performed with endoscopic scissors from the vaginal endoscope. A laparoscopic stapler closed the common enterotomy. The pouch was formed using a stapler through the right vaginal port. A gastro-enteric anastomosis was formed using a stapler inserted orally and an anvil inserted transvaginally. The vagotomies were left open.

In all subjects, the resected small bowel was removed through the vaginal opening [10, 12].

### Endolumenal Procedures

In addition to NOTES procedures, there are also studies indicating the use of endolumenal restrictive procedures for weight loss. The majority of these studies use a technique referred to as either gastroplasty or gastric plication. These procedures involve the use of either sutures or staples to reduce the lumen size of the stomach.

Table 6 outlines the available literature on these procedures alongside some of the available data.

**Table 6:** Endoscopic restrictive procedures in obese patients

Author	Year of publication	Number of subjects	Surgical technique	Mean operative time: min (range)	Serious postoperative complication rate
Abu Dayyeh et al. [19]	2013	4	Transoral sleeve gastroplasty	N/A (172–245)	Nil
Brethauer et al. [20]	2010	18	TRIM endoluminal gastric plication	125 +/− 23	Nil
Devière et al. [21]	2008	21	Transoral vertical gastroplasty	131	Nil
Fogel et al. [22]	2008	64	Endoluminal vertical gastroplasty	45 *approximated*	Nil
Moreno et al. [23]	2008	11	Transoral vertical gastroplasty	84	Nil
Nanni et al. [24]	2012	29	Transoral vertical gastroplasty	70 (55–115)	Nil

### Clinical Outcomes

There were no serious complications stated by any of the publications analysed. However, postoperative pain was a well-documented adverse outcome. Devière et al. [19] found this to be the case in 13 out of 21 patients. Whilst Moreno et al. [20] found all 11 patients experienced epigastric pain after the procedure. Interestingly, whilst not describing the same patients, these two papers were from the same group—using the TOGa system.

Mean operative time of the studies is incalculable. As such, it is not possible to draw comparisons to NOTES procedures.

The hospital stay in these patients was significantly reduced compared to hNSG. Disregarding the Devière and Moreno papers, the other participants in the other studies [21–24] were either discharged the same day, or kept overnight for observation only. There was one exception [21] where a patient was admitted for 72 h with abdominal pain that resolved with conservative management.

Mean excess weight loss was observed in four studies. These ranged from 24.4 to 46.0 % loss, at 6 months [19, 20].

### Surgical Technique

The TOGa system was utilised in three studies with no differences in technique noted [19, 20, 24]. Devière et al. [19] placed patients supine and used an upper endoscope to locate the Z-line. A guide wire was placed into the stomach and the endoscope was subsequently removed. A 60 Fr bougie was then introduced over the guide wire. The TOGa sleeve stapler was introduced over the guide wire into the stomach. A smaller endoscope was introduced using a channel in the stapler for direct visualisation of the procedure. Suction was employed by the device in order to draw anterior and posterior tissue into two vacuum pods. The stapler was subsequently discharged producing three rows of 11 staples. This was repeated to insert another staple line. This created a 19-mm diameter sleeve with an outlet that was constricted to 10–12 mm using the TOGa restrictor.

Abu Dayyeh et al. [21] utilised sutures as opposed to staples. An overtube was inserted down the oesophagus before identifying anterior and posterior suture sites. Three linear opposing interrupted sutures were placed from the pre-pyloric antrum up to the GOJ in order to reduce the gastric lumen. The sutures were inserted using the Overstitch device that uses a helix to capture the full-thickness of the gastric tissue—both anteriorly and posteriorly. A cinching device was used to complete the plication. Approximately 10 interrupted sutures were then placed to reduce the gastric body. The fundus is closed using two layers of up to five opposing sutures. A final plication was located at the GOJ.

Brethauer et al. [22] used the Restore Suturing System for transoral gastric volume reduction (TRIM procedure). The device works using a capsule at the end of a standard endoscope that employs applies suction. The system itself is used through the working channel of the endoscope. A range of four to eight plications were made in each procedure. The first plication, placed proximally along the greater curvature, decreased the fundus size. Another was placed even more proximally to further reduce size. Three bite plications were made 10 cm distally to the GOJ. Final plications moved proximally towards the angle of His.

Fogel et al. [23] conducted an endolumenal vertical gastroplasty in 64 patients. For this, they used the Bard EndoCinch Suturing System. Initially, a 45-Fr overtube was advanced over a guide wire, before removal of said guide wire and insertion of the endoscope. One continuous suture was inserted in the gastric wall consisting of 5 to 7 bites. The first bite was taken in the most proximal folds on the anterior face of the fundus, whilst the second was placed on the most distal rugae of the anterior surface. The third stitch was made on the posterior surface 1 to 2 cm proximally to the second. The subsequent stitches alternated anteriorly and posteriorly towards the first stitch, with the final one sited on the posterior surface—1 to 2 cm proximally to the first. The suture was then tightened and secured to reduce the lumen size.

## Discussion

NOTES has gained popularity during recent years for less complex procedures such as cholecystectomy and appendicectomy. This is supported by evidence that when performed by appropriately trained surgeons the results can be favourable to that of conventional laparoscopic technique [7, 25].

There is a paucity of data in relation to the clinical applications of bariatric NOTES procedures. The majority of data in the literature relates to sleeve gastrectomies, which appears technically to be the most feasible. The clinical outcomes seem promising, with limited morbidity from the published series. The obvious issue that needs to be overcome is the limited assistance provided by the transvaginal port. Many studies were unable to perform key steps such as division of short gastric vessels and transection of the stomach through this port, limiting further minimisation of ports. There were several reasons suggested for these difficulties. These included, but were not limited to the following: length of stapler and energy delivery system, as well as ergonomic challenges such as rigid instrumentation and distance to target—a particular issue in this subset of patients.

Other procedures have proven more difficult. For hNAGB, the major difficulty lies with being able to position and fasten the band, even with laparoscopic assistance with 33.3 % of patients having to be reverted to traditional laparoscopic placement. In Roux-en-Y gastric bypass, the necessity of opening the lumen of the bowel would suggest that it is a perfect candidate for NOTES adaptation. However, the operative complexity is great with this procedure and difficulties were noted in particular with gastric pouch formation as well as entero-entero anastomosis.

Endolumenal procedures are promising in terms of feasibility. These procedures provide a theoretical advantage by enabling the operator to perform a restrictive procedure, without the need for the incision required by NOTES. This could be particularly beneficial in regard to removing potential concerns associated with closure of the orifice, with the additional benefit of removing the difficulty associated with current endoscope length. However, there is great heterogeneity of techniques and outcome data is limited. More data is necessary to compare clinical outcomes in particular long-term weight loss, re-operations and resolution of comorbidities. In theory, these techniques are the least invasive, but more data is needed to suggest their clinical applicability. The future of endolumenal procedures seems to lie with the use of restrictive procedures to reduce gastric volume. Again, further data is needed to validate a device, which provides the greatest outcomes, along with the overall clinical applicability of endolumenal procedures.

In examining NOTES in the full context of all types of scarless surgery, one must consider the role of SILS in bariatric procedures. SILS, despite being more recognised, is still not recommended for most patients over traditional multi-port surgery [26]. The aim with scarless surgery should be to provide patients with the most clinically effective treatment at the lowest risk of morbidity or mortality, not purely a reduction in the number of port sites. However, obese patients are always likely to provide greater challenges—hindering the uptake of increasingly minimally invasive techniques.

Currently, there is a lack of a specific NOTES operating platform that will allow more complex operative manoeuvres. Using the standard flexible endoscope and conventional laparoscopic instruments limits the complexity of operations that can be performed. Based on this data, Roux-en-Y gastric bypass is too complex to be performed as a NOTES procedure.

Further research should focus on developing procedure specific NOTES platforms that are flexible, provide stability and have advanced capabilities including stapling and suturing. To overcome these issues is dependent on creating a fully articulated device [27]. This would ensure that there is adequate triangulation of instruments to improve operative outcomes, whilst also reducing disorientation. It is clear, however, that these advanced techniques are directly linked to technological innovation for which we are reliant on industry for the further development.

In conclusion, hNSG appears most feasible in the medium-term aided by the introduction of flexible stapling and energy devices. Further research should focus on engineering solutions and specific platforms tailored for NOTES. Moreover, larger clinical cohort studies are required to confirm the feasibility of the technique and to compare clinical outcomes of the procedure in both the short and long-term.
